# Raman Spectroscopy for Early Detection of Cervical Cancer, a Global Women’s Health Issue—A Review

**DOI:** 10.3390/molecules28062502

**Published:** 2023-03-09

**Authors:** Rubina Shaikh, Amuthachelvi Daniel, Fiona M. Lyng

**Affiliations:** 1 Centre for Radiation and Environmental Science, FOCAS Research Institute, Technological University Dublin—City Campus, D02 HW71 Dublin, Ireland; 2 School of Physics, Clinical and Optometric Sciences, Central Quad, Technological University Dublin—City Campus, D07 XT95 Dublin, Ireland; 3 Timegate Instruments Ltd., 90590 Oulu, Finland

**Keywords:** cancer screening, human papillomavirus, cervical cancer, Raman spectroscopy, vibrational spectroscopy, exfoliated cells, tissues, biofluid, in vivo, ex vivo

## Abstract

This review focuses on recent advances and future perspectives in the use of Raman spectroscopy for cervical cancer, a global women’s health issue. Cervical cancer is the fourth most common women’s cancer in the world, and unfortunately mainly affects younger women. However, when detected at the early precancer stage, it is highly treatable. High-quality cervical screening programmes and the introduction of the human papillomavirus (HPV) vaccine are reducing the incidence of cervical cancer in many countries, but screening is still essential for all women. Current gold standard methods include HPV testing and cytology for screening, followed by colposcopy and histopathology for diagnosis. However, these methods are limited in terms of sensitivity/specificity, cost, and time. New methods are required to aid clinicians in the early detection of cervical precancer. Over the past 20 years, the potential of Raman spectroscopy together with multivariate statistical analysis has been shown for the detection of cervical cancer. This review discusses the research to date on Raman spectroscopic approaches for cervical cancer using exfoliated cells, biofluid samples, and tissue ex vivo and in vivo.

## 1. Introduction

### 1.1. Cervical 

According to GLOBOCAN [1], cervical cancer is the fourth most common cancer in women worldwide in terms of incidence and mortality, with 604,127 new cases and 341,831 deaths in 2020 [1]. Persistent infection with high-risk human papillomavirus (HPV) is accepted as the major cause of the development of cervical cancer and precancer [2]. Over 100 different types of HPV have been identified, and 14 are considered to be high-risk HPV types (hrHPV 16, 18, 31, 33, 35, 39, 45, 51, 52, 56, 58, 59, 66, and 68) [3]. Smoking, immunosuppression, long-term use of oral contraceptives, and socioeconomic status are also known risk factors for cervical cancer [4]. 

In 2018, the World Health Organisation launched a global initiative to eliminate cervical cancer through three main actions: HPV vaccination, cervical cancer screening, and effective treatment [5]. At present, three HPV vaccines are available: (1) a bivalent vaccine targeting high-risk HPV16 and HPV18 (accounting for ~70% of cervical cancer cases), (2) a quadrivalent vaccine targeting HPV16, HPV18, and low-risk HPV6 and HPV11, and (3) a nonavalent vaccine targeting HPV16, HPV18, HPV6, HPV11, and five other high-risk types, HPV31, HPV33, HPV45, HPV52, and HPV58 (accounting for another 20% of cervical cancer cases) [6]. Despite these vaccines having excellent efficacy against cervical precancer lesions [6,7], vaccination levels are low in low- and middle-income countries, which carry the burden of 80% of global cancer cases [8]. Moreover, high-quality cervical screening programmes are still essential, as HPV vaccination does not protect against all high-risk HPV types [9]. 

### 1.2. Cervical Cancer Screening and Diagnosis

For decades, cytology was used for primary cervical screening, but recently, HPV testing has replaced cytology in many countries because HPV testing has a higher sensitivity than cytology for the detection of high-grade cervical precancer (cervical intraepithelial neoplasia (CIN)2+) [10]. Additional triage tests are required, however, for HPV-positive women, because HPV testing has a lower specificity than cytology [11]. Cytology triage is currently utilised to reduce over-referral to colposcopy and overtreatment of HPV-positive women.

Following an abnormal Pap test (HPV test with cytology triage), a woman will be sent for colposcopy to visualise the cervix followed by a tissue biopsy and histopathology. Histopathology is currently regarded as the ‘gold standard’ test for the diagnosis of cancer but the grading characteristics can be subjective and precancer may not be visually perceptible. Current methods for screening and diagnosis of cervical cancer and precancer are therefore limited, and there has been much interest in the use of optical spectroscopic approaches, such as Raman spectroscopy, to provide an objective test based on the biochemical fingerprint of the cervical cells or tissues.

### 1.3. Raman Spectroscopy

Raman spectroscopy is based on inelastic scattering, which has been used to study the biomolecular fingerprint of cells or tissues [12]. It involves shining a laser on a sample and measuring the scattered photons. When a photon collides on a molecule, it either retains its energy (known as Rayleigh scattering) or exchanges energy with the molecule (known as Raman scattering). The resulting Raman spectra are plots of scattered intensity versus the energy difference between incident and scattered photons, and exhibit changes in the vibrational modes of the molecules. Raman spectra are characterized by shifts in wavenumbers (inverse of wavelength in cm^−1^) from the incident frequency. The frequency difference between incident and scattered Raman photons is termed the Raman shift, which is unique to any given molecule.

### 1.4. Evolution of Raman Spectroscopy and Its Application in Cervical Cancer

**Figure 1:**
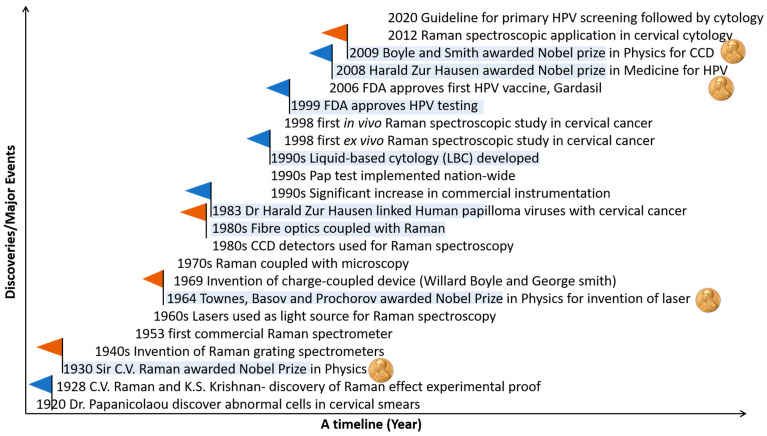
Major events in cervical cancer screening and Raman spectroscopy (blue colour––flag event in Cervical cancer screening; orange colour––flag events in technological advancement in Raman spectroscopy).

During a sea voyage from India to England in 1921, the Indian Physicist C.V. Raman conducted experiments, later submitted to Nature as a letter called ‘The Colour of the Sea’ [13] (Figure 1). A later series of experiments on the scattering of light in different liquids led to the discovery of the inelastic scattering effect named after Raman in 1928 [14], and this discovery was awarded the Nobel Prize in Physics in 1930. One of the known limitations of Raman spectroscopy was, for example, a weak scattering [12]: typically 1 in 10^8^ of the incident light undergoes spontaneous Raman scattering. Additional limitations were long spectral acquisition time, background fluorescence, and interference from silica in fibre optics [12]. Many of these limitations were overcome by the invention of lasers in the 1960s, and fibre-optic probes and CCD in the 1980s [12]. 

In parallel with the technological advances in the field of Raman spectroscopy, there was development in the field of cervical cancer and its screening methods. For example, Dr Papanicolaou and Dr Herbert collaborated on the diagnosis of uterine cancer using the vaginal smear in August 1943 [15], and the current cervical screening has evolved from this cervical screening test, popularly known as the Papanicolaou (Pap) test [16] (Figure 1). Later, the human papillomavirus virus (HPV) was linked to cervical cancer, leading to the Nobel Prize [17]. Nationwide screening using the Pap test was implemented in 1990 in developed nations, resulting in a >70% decline in both the incidence and mortality of cervical cancer [18].

In the 1990s, technological advancements in cytology led to the development of liquid-based cytology (LBC), which was shown to be of better quality than the conventional Pap test [16]. Multiple studies have exhibited a significant impact of LBC in decreasing the incidence of cervical cancer [19]. The 2000s saw the emergence of a HPV vaccine [20] and other revolutions, such as co-testing to reduce the cervical cancer burden [21]. In recent years, HPV-based screening with cytology has been implemented in the Netherlands, Germany, Italy, the UK, and Ireland [22,23]. Contrary to this, developing nations struggle to have national-level cervical screening programmes, either due to a lack of infrastructure or trained histopathologists [22]. To date, cervical cancer remains a major global challenge. 

There have been previous efforts at this by multiple authors, either by meta-analysis [23] or by an overview of the field [24,25,26,27,28,29,30]. This review will present the research to date on Raman spectroscopic approaches for cervical cancer using exfoliated cells, tissue ex vivo and in vivo, and biofluid samples, and it will discuss future directions in the post-vaccination era. 

## 2. Cytology

Table 1 summarises the studies to date on the application of Raman spectroscopy in cervical cytology.

### 2.1. Cell Pellets

Vargis et al. [31] demonstrated that Raman spectroscopy could detect the presence of high-risk HPV in cytology samples. Spectral differences were observed in regions corresponding to lipid, amino acid, and deoxyribonucleic acid (DNA) content as well as CH stretching and bending regions assigned to proteins. HPV-positive and HPV-negative cytology samples were discriminated with an accuracy of 98.5%. 

Rubina et al. [32] used Raman spectroscopy to discriminate between exfoliated cell pellets from patients with negative cytology and cervical cancer cytology. Classification efficiency of ~90% was achieved using principal component analysis-linear discriminant analysis (PCA-LDA) [33], but heme and fibrin bands from blood appeared to be major discriminating features. A subset of cell pellets was treated with red blood cell lysis buffer before Raman spectroscopy. Successful blood removal was confirmed by the absence of heme and fibrin bands, and the main discriminating feature was an increase in protein content in the cervical cancer samples compared to the negative samples. A classification efficiency of ~80% was achieved using PCA-LDA. Misclassifications were attributed to sample heterogeneity and the predominance of normal cells in the cervical cancer samples. 

This work was extended by Hole et al. [34] to Raman analysis of cervical and oral exfoliated cell pellets. The main discriminating features were DNA and protein, and improved classification was achieved when mean spectra from each sample were used to overcome intra-sample heterogeneity (84% cervical and 86% oral cancer) compared to all spectra from each sample (77% cervical and 82% oral cancer).

**Table 1:** Application of Raman spectroscopy in cervical cytology.

Year	Authors (Research Group)	Sample/Patient Numbers	Sample Type/Sample Prep/Substrate	Raman Parameters	Data Analysis Methodology	Main Findings	Reference
2012	Vargis et al. (Mahadevan-Jansen group)	50 patient samples—25 HPV negative and 25 HPV positive	Centrifugation and washing with sterile water Pellets of exfoliated cells on calcium fluoride slides	Renishaw Invia Raman microscope 785 nm laser, ∼30 mW at the sample 50X/0.75 NA objective lens Spectral resolution ∼6 cm^−1^ 45 to 60 s acquisition, 3 accumulations	Sparse multinomial logistic regression (SMLR)	Discrimination of HPV-positive and HPV-negative cytology samples Spectral differences: lipid, amino acid, protein, and DNA content Accuracy: 98.5%	[31]
2013	Rubina et al. (Krishna group)	94 patient samples—45 negative cytology and 49 cervical cancer cytology	Centrifugation and washing with saline Pellets of exfoliated cells on calcium fluoride slides Red blood cell (RBC) lysis buffer treatment	Horiba- Jobin-Yvon fibre-optic Raman microprobe system 785 nm laser ∼40 mW at the sample 40X/0.65 NA objective lens Spectral resolution ∼4 cm^−1^ 6 s acquisition, 3 accumulations	Principal Component Analysis Linear Discriminant Analysis (PCA-LDA)	Treatment of cell pellet with RBC lysis buffer to remove blood contamination Discrimination of negative and cancer cytology Spectral differences: protein Accuracy: 80%	[32]
2014	Bonnier et al. (Lyng group)	63 patient samples—50 negative cytology and 13 high-grade cytology	ThinPrep method Single exfoliated cells on ThinPrep glass slides Hydrogen peroxide pre-treatment	Horiba- Jobin-Yvon XploRA Raman microscope 532 nm laser ∼8 mW at the sample 100X/0.9 NA objective lens Spectral resolution ∼3 cm^−1^ 10 s acquisition, 3 accumulations	PCA	Pre-treatment of ThinPrep slides with hydrogen peroxide to eliminate variability due to blood contamination Discrimination of negative and high-grade cytology Spectral differences: DNA, RNA	[35]
2016	Ramos et al. (Lyng group)	166 patient samples—88 negative cytology, 35 low-grade cytology, and 43 high-grade cytology	Sample preparation as for Bonnier et al. [28]	Raman set up as for Bonnier et al., 30 s acquisition, 2 accumulations	PCA-LDA	Discrimination of negative, low-grade, and high-grade cytology Spectral differences: lipids, nucleic acids, and proteins Sensitivity: 90.91–100% Specificity: 97.24–100%	[36]
2017	Kearney et al. (Lyng group)	80 patient samples—30 negative cytology, 50 high-grade cytology	Sample preparation as for Bonnier et al. [28]	Raman set up as for Bonnier et al., 30 s acquisition, 2 accumulations	PCA-Factorial Discriminant Analysis (FDA)	Raman spectral signatures of superficial, intermediate, and parabasal cells High variability in spectra from cytoplasm due to glycogen Discrimination of negative and high-grade cytology Spectral differences: lipids, nucleic acids and proteins Sensitivity: 92%, Specificity: 97%	[37]
2018	Traynor et al. (Lyng group)	60 patient samples—45 negative cytology and 15 high-grade cytology	Sample preparation as for Bonnier et al. [28]	Raman set up as for Bonnier et al., 30 s acquisition, 2 accumulations	PLSDA	Biochemical changes due to high-grade cytology more pronounced than hormone related changes Discrimination of negative and high-grade cytology Spectral differences: glycogen, nucleic acids, and proteins Sensitivity: 96–98%, Specificity: 97–98%	[38]
2018	Duraipandian et al. (Lyng group)	35 patient samples—18 negative cytology and 17 high-grade cytology	Sample preparation as for Bonnier et al. [28]	Raman set up as for Bonnier et al., 30 s acquisition, 2 accumulations	PCA-LDA and PLSDA	Discrimination of negative and high-grade cytology (morphologically normal superficial and intermediate cells) Spectral differences: glycogen, nucleic acids, and proteins Sensitivity: 75.6% (PCA-LDA), 96.1% (PLSDA) Specificity: 84.5% (PCA-LDA), 93.5% (PLSDA)	[39]
2018	Traynor et al. (Lyng group)	30 patient samples—15 negative cytology and 15 high-grade cytology	Sample preparation as for Bonnier et al. [28]	Raman set up as for Bonnier et al. 30 s acquisition, 2 accumulations	PLSDA	Pre-treatment of ThinPrep vial with hydrogen peroxide to remove excessive blood contamination (blood scale index 2–3) Discrimination of negative and high-grade cytology (morphologically normal superficial and intermediate cells) Spectral differences: glycogen, nucleic acids, and proteins Sensitivity: 82–92%, Specificity: 87–93%	[40]
2018	Hole et al. (Krishna group)	66 patient samples—28 negative cytology and 38 cervical cancer cytology	Centrifugation and washing with saline Pellets of exfoliated cells on calcium fluoride slides Red blood cell (RBC) lysis buffer treatment	Horiba-Jobin-Yvon fibre-optic Raman microprobe system 785 nm laser ∼40 mW at the sample 40X/0.65 NA objective lens Spectral resolution ∼4 cm^−1^ 15 s acquisition time, 3 accumulations	PCA-LDA	Discrimination of negative and cancer cytology Spectral differences: DNA, protein Accuracy: 84%	[34]
2019	Aljouch et al. (El-Mashtoly/Gerwert group)	30 patient samples—10 negative, 10 low-grade and 10 high-grade cytology	Single exfoliated cells prepared using a cytospin centrifuge onto quartz slides	WITec Raman microscope 532 nm laser 60X/1.0 NA objective lens, water immersion	Deep convolutional neural networks (DCNN)	Raman imaging of single exfoliated cells Discrimination of negative, low-grade, and high-grade cytology Spectral differences: lipids, proteins, polysaccharides, and nucleic acids Accuracy: 94–100%	[41]
2020	X. Zheng et al. (Wu group)	63 patient samples 33-normal 30- HR-HPV	5 µL of preserved cell samples on aluminum foil, dried at room temperature	LabRam HR evolution with 532 nm laser source was focused using 50X objective	PCA-LDA	The authors observed the diagnostic accuracy of 99.4%	[42]
2020	Sitarz et al. (Kaczor group)	96 patient samples––negative, low-grade, high-grade, and cancer cytology and HPV- and HPV+	Single exfoliated cells fixed with 2.5% glutaraldehyde, washed in PBS, and placed on calcium fluoride slides	WITec Raman microscope 532 nm laser ∼28 mW at the sample 63X/1.0 NA objective lens, water immersion Spectral resolution ∼3 cm^−1^	Cluster analysis (CA)	Increased glycogen metabolism with HPV infection—for cells with large nuclear diameter, glycogen decreased in HPV positive compared to HPV negative samples	[43]
2020	Karunakaran et al. (Maiti group)	124 patient samples comprising 47 negative, 41 high-grade, and 36 cancer cytology	Single exfoliated cells, cell pellets and extracted DNA incubated with gold nanoparticles (AuNPs, 40–45 nm) on glass slide	WITec Raman microscope 633 nm laser 20X objective lens	Support vector machines (SVM)	Discrimination of negative, high-grade, and cancer cytology Spectral differences—nucleic acids and amino acids Accuracy: 94.46% (single exfoliated cells), 71.6% (cell pellets) and 97.72% (extracted DNA)	[44]
2021	Karunakaran et al. (Maiti group)	9 patient samples comprising 3 negative, 3 high-grade, and 3 cancer cytology	Density gradient centrifugation Single exfoliated cells on glass slide Incubation with SERS nanotag for 45 min	WITec Raman microscope 633 nm laser 10 mW power 20X objective lens Spectral resolution ∼1 cm^−1^ 10 s acquisition, 3 accumulations	n/a	SERS detection of cervical cancer biomarkers p16 and Ki67 in single exfoliated cells	[45]
2021	Sitarz et al. (Kaczor group)	63 patient samples comprising negative, low-grade, high-grade, and cancer cytology and HPV- and HPV+	Sample preparation as for Sitarz et al. [36]	Raman set up as for Sitarz et al.	K means cluster analysis (KMCA)	Dual switch of lipid metabolism—decreased lipid in low-grade cytology and increased lipid in high-grade and cancer cytology compared to negative cytology	[46]
2021	Traynor et al. (Lyng group)	60 patient samples for training set—30 HPV DNA positive, mRNA negative and 30 HPV DNA positive, mRNA positive 14 blinded patient samples for test set	Sample preparation as for Bonnier et al. [28]	Raman set up as for Bonnier et al. 30 s acquisition, 2 accumulations	PLSDA	Discrimination of transient and transforming HPV infections (morphologically normal superficial and intermediate cells) Spectral differences: glycogen, nucleic acids and proteins Accuracy: 93%	[47]
2022	Traynor et al. (Lyng group)	662 patient samples for training set—326 negative cytology, 200 low-grade cytology and 136 high-grade cytology 69 blinded patient samples for test set	Sample preparation as for Bonnier et al. [28]	Raman set up as for Bonnier et al. 30 s acquisition, 2 accumulations	PLSDA	Discrimination of negative, CIN1, and CIN2+ samples (morphologically normal superficial and intermediate cells) Spectral differences: glycogen, nucleic acids, and proteins Accuracy: 91.3%	[48]

n/a: not applicable; no multivariate analysis was carried out in the study.

### 2.2. Single Exfoliated Cells

In contrast to earlier work on cellular pellets, Bonnier et al. [35] presented a new method for recording Raman spectra from cervical cytology samples prepared as single exfoliated cells using the ThinPrep liquid-based cytology method. Pre-treatment of the slides with a hydrogen peroxide solution to clear blood residue contamination before Raman recording was shown to significantly minimize variability in the spectral data. Features related to the DNA/RNA content of the cells contributed to the discrimination of spectra from negative cytology samples and high-grade cytology samples. This pre-treatment method was later adapted to Thinprep specimens with excessive blood contamination (blood scale index 2–3) by adding hydrogen peroxide directly to the vial before slide preparation [38]. 

In this study on Raman spectra from negative cytology and high-grade cytology samples after pre-treatment, glycogen, nucleic acids, and proteins were found to be the main discriminating features regardless of whether the samples had minimal blood contamination (blood scale index 0) or excessive blood contamination (blood scale index 2–3). Traynor et al. [25] published a protocol for Raman spectral cytopathology for use on liquid-based cytology samples prepared onto glass slides. This protocol covered sample preparation, spectral acquisition, pre-processing, and data analysis, and it included methods of correction of the glass spectral contribution and sample pre-treatment methods to remove contaminants, such as blood and mucus. 

Using the same protocol, Ramos et al. [36] investigated if Raman spectroscopy could be applied to routine cervical cytology samples from a cervical screening programme. Raman spectra were recorded from ThinPrep samples with negative, low-grade, and high-grade cytology. Cell nuclei from normal cells (negative cytology samples) and abnormal cells (low-grade and high-grade cytology samples) were targeted. Protein features were the main discriminating feature, although some differences in nucleic acid features were also observed. A change in ratio at 1318/1339 cm^−1^ was also observed for negative, low-grade, and high-grade cases, suggesting a decrease in the lipid/protein to guanine ratio in low-grade and high-grade cytology samples, either as a result of a reduction of lipids/proteins and/or an increase in nucleic acid (guanine) content. Sensitivity and specificity values > 90% were achieved when the cervical intraepithelial neoplasia (CIN) terminology was used to classify the samples compared to the squamous intraepithelial lesion (SIL) terminology.

Kearney et al. [37] continued this work on single exfoliated cells and defined the Raman spectral signatures of superficial, intermediate, and parabasal cells, which are the main cell types present in liquid-based cytology Pap test specimens. Raman spectra were recorded from both the nuclei and from the cytoplasm of negative cytology and high-grade cytology samples, and the spectra from the cytoplasm showed significant variability, which was shown to be due to different levels of glycogen in the cells at different phases of the menstrual cycle. Glycogen, nucleic acids, and proteins were the main differentiating features between Raman spectra from the nuclei of cells with normal cytology and those with high-grade cytology. A further investigation of hormone-associated variability related to the menstrual cycle, menopause, and the use of hormone-based contraceptives on the Raman spectra was carried out by Traynor et al. [40] on negative cytology and high-grade cytology ThinPrep samples. The findings showed that post-menopausal samples could be problematic for Raman spectral analysis due to a lack of cellular material and the presence of cellular debris and mucus. In addition, although hormone-related spectral changes in glycogen and protein features were observed, it was found that biochemical changes in cells with high-grade cytology were more pronounced than biochemical changes in cells due to the menstrual cycle or the use of hormone-based contraceptives. 

Up to this point, abnormal cells with high-grade cytology had been investigated using Raman spectroscopy. To overcome the challenge associated with finding the rare abnormal cells on the unstained ThinPrep slide, Duraipandian et al. [39] showed that biochemical differences between negative cytology and high-grade cytology samples could be detected in cells that appear normal. 

Using morphologically normal single exfoliated cells, Raman spectroscopy was investigated as a potential triage test to discriminate between transient and transforming HPV infections [47]. HPV, DNA, and mRNA testing were carried out, and Raman spectra were recorded from single cell nuclei. Discrimination was mostly based on increased nucleic acids (727, 781, 826, 1485, and 1580 cm^−1^), decreased glycogen (482, 852, 937, 1082, 1123, 1334, and 1380 cm^−1^), and changes in protein features (1152, 1240, 1450, 1640, and 1670 cm^−1^), indicating increased proliferation and altered protein expression due to the overexpression of E6/E7 viral proteins. A PLSDA classification model was trained using 60 ThinPrep cervical samples and then validated using a blinded independent test set of 14 ThinPrep cervical samples, achieving an accuracy of 93%.

Again using morphologically normal single exfoliated cells, Traynor et al. [48] investigated the clinical utility of Raman spectroscopy for identifying cervical precancers in a large sample set of 662 ThinPrep cervical samples. Raman spectra were recorded from single cell nuclei of negative, CIN1, and CIN2+ samples as a training set. A PLSDA classification model was validated using a blinded independent test set of 69 ThinPrep cervical samples, achieving an accuracy of 91.3%.

### 2.3. Raman Imaging

Coherent anti-Stokes Raman scattering (CARS)/second harmonic generation (SHG)/two-photon excited autofluorescence (TPF) imaging followed by Raman imaging has been applied to liquid-based Pap smear samples [41]. The main discriminating features were lipids, proteins, polysaccharides, and nucleic acids, and deep convolutional neural networks (DCNNs) achieved 100% accuracy for the classification of negative, low-grade, and high-grade cytology based on Raman spectral data and on morphological features obtained from CARS/SHG/TPF images.

Sitarz et al. [43] used Raman imaging to assess glycogen levels in the cytoplasm of cervical exfoliated cells. For cervical epithelial cells with small diameter nuclei, glycogen content was similar for HPV-negative and HPV-positive samples, whereas, for cells with large diameter nuclei, glycogen content decreased in HPV-positive compared to HPV-negative samples, indicating that glycogen metabolism is accelerated with HPV infection. A follow-on study from the same group investigated the lipid profile of cervical exfoliated cells [46]. Lipid content was found to decrease in samples with low-grade cytology and increase in samples with high-grade cytology compared to samples with negative cytology, suggesting a dual switch of lipid metabolism.

### 2.4. SERS

A SERS approach has been applied to cervical exfoliated cells to discriminate normal, high-grade precancer, and cervical squamous cell carcinoma [44]. Gold nanoparticles were used to enhance the Raman signal, and spectra were recorded from single exfoliated cells, cell pellets, and extracted DNA. Nucleic acids and amino acids were the main discriminating features. Classification accuracies of 94.46%, 71.6%, and 97.72% were achieved for single exfoliated cells, cell pellets, and extracted DNA, respectively. For extracted DNA, high accuracy was achieved for normal, high-grade precancer, and cancer samples, whereas for the single exfoliated cells and cell pellets, high accuracies were achieved for normal and cancer samples but not for high-grade samples. This SERS approach was further extended to the simultaneous detection of cervical cancer biomarkers, p16 and Ki67, in single exfoliated cells using a SERS-tag functionalized with the monoclonal antibodies against p16/Ki67 [45].

## 3. Ex Vivo Studies on Cervical Tissues

Table 2 summarises the studies to date on the application of Raman spectroscopy in cervical tissues (ex vivo).

The first near-infrared Raman spectroscopy study for ex vivo detection of cervical precancer was reported in 1998 as a proof of concept before moving to in vivo fibre-optic Raman spectroscopy [49]. Later, two reports studied the prediction of radiation response in cervical cancer via ex vivo Raman spectroscopy [50,51]. The results showed that Raman spectroscopy can predict if the patient responds to radiotherapy or not as early as the second fraction of radiotherapy [50,51].

The Raman micro-spectroscopic mapping study by Rashid et al. showed that the normal cervical tissue’s spectral features of collagen, DNA bases, and glycogen differentiated three different layers––stromal, basal, and intermediate/superficial layers [52]. The authors reported that principal component analysis (PCA) showed biochemical differences between morphologically normal tissue of the abnormal (HSIL) and normal (NILM) samples [52]. A study by Daniel et al. characterizing cervical tissue sections by polarized and conventional Raman spectroscopy concluded that polarized Raman spectroscopy yielded better classification accuracy [53]. Zheng et al. showed Raman spectral differences between cervical tissue from adenocarcinoma and squamous cell carcinoma [54]. In 2021, two studies from the Lv group characterized cervical tissue using Raman spectral feature extraction combined with various machine learning algorithms [55,56], and Wang et al. developed models to classify cervicitis, CIN I, CIN II, CIN III, cervical squamous cell carcinoma, and cervical adenocarcinoma using support vector machines with an overall diagnostic accuracy of 85.7% [57]. 

**Table 2:** Application of Raman spectroscopy in cervical tissues (ex vivo).

Year	Authors (Research Group)	Sample/Patient Numbers	Sample Type/Sample Prep/Substrate	Raman Parameters	Data Analysis Methodology	Main Findings	Reference
1998	Mahadevan-Jansen et al. (Rebecca Richard-Kortum’s group)	36 biopsies from 18 patients	Tissues snap-frozen in liquid nitrogen and stored at −85 ºC, until measurements	A 40 mW GaAlAs diode laser (Diolite 800, LiCONix, Santa Clara, CA, USA) was used to excite samples at 789 nm through a 200 Wm core diameter glass optical fibre	PCA	Discrimination of inflammation and metaplasia from squamous intra-epithelial lesions	[49]
2008	Vidyasagar et al. (Krishna group)	25 malignant tissues, 25 samples after radiotherapy and 16 normal tissues	Tissue samples collected in saline.	In house-built Raman setup, which used 785 nm laser source and HR 300 spectrograph coupled to liquid nitrogen-cooled CCD was used.	PCA	Classification between responding and nonresponding tissues.	[50]
2013	Rubina et al. (Krishna group)	11 normal, 16 tumor, 14 post radiation	Tissues were collected in PBS and stored in liquid nitrogen.	LabRam equipped with a 785 nm laser source and a spectrograph with 950 groves/mm and CCD as detector was used.	PC-LDA	Differentiation between pre- and post-treated tumor tissues.	[51]
2014	Rashid et al. (Lyng group)	5 NILM, 2LSIL, 10 HSIL (5 CIN 2, 5 CIN 3) and 3 carcinomas in-situ	10-micron thickness formalin fixed paraffin cervical tissue was cut and mounted on a calcium fluoride slide and dewaxed.	Horiba Jobin Yvon HR800 Raman microscope with a 785 nm laser source, 100X air objective. The spot size was ~1 micron on the sample, the confocal hole was 100 microns on a CCD detector for the range between 400–1800 cm^−1^ using 300 lines/mm diffraction grating	KMCA and PCA	Differentiation of NILM cervical tissue into three layers—stroma, basal, and superficial layers. For HSIL tissue with normal and abnormal regions, the normal region was not normal as in the case of NILM samples.	[52]
2015	Daniel et al. (Ganesan group)	36 normal and 25 cervical cancer samples	Fresh frozen tissues sliced to 20-micron thickness were mounted on a quartz slide.	LabRam HR 800, 784.12 nm laser was focused using a 50X objective, Polarized Raman spectra were obtained by placing an analyzer along the parallel and perpendicular	LDA	Polarized Raman spectroscopy provided better classification.	[53]
2019	Zheng et al. (Yue group)	95 cases- 45 cervical adenocarcinomas 50 cervical squamous cell carcinomas	10-micron FFPP tissue sections were dewaxed.	LabRam HR evolution with 532 nm laser source was focused using 50X objective, 100 um step-size	PCA-SVM model	Differentiation between cervical adenocarcinoma and cervical squamous cell carcinoma	[54]
2021	Zhang et al. (Lv group)	49 inflammation samples, 29 LSIL samples and 45 HSIL samples.	Formalin-fixed tissue embedded in paraffin, cut into 10-micron thick tissue sections and dewaxed	Labram HR evolution using an excitation wavelength of 532 nm and focused using a 50X objective lens was employed, the laser spot at the sample was 6 microns, and the power on the sample surface was 100 mW.	airPLS-PLS-KNN and airPLS-PLS-ELM; feature fusion-KNN and feature fusion-ELM.	Accuracy of the model increased by 5.38% and 2.7% after feature fusion.	[55]
2021	Yang et al. (Lv group)	45 cervicitis samples, 29 LSIL, 44 HSIL, 39 SCC and 38 adenocarcinoma samples.	Formalin-fixed tissue embedded in paraffin, cut into 10-micron thick tissue sections and dewaxed	LabRam HR evolution with an excitation wavelength of 532 nm was used.	KNN, ELM, ABC-SVM, CS-SVM, PSO-SVM and CNN-LSTM	Classification of cervicitis, LSIL, HSIL, SCC and adenocarcinoma	[56]
2021	Wang et al. (Wu group)	210 samples 60-Cervicitis, 30-CIN I, 30-CIN II, 30-CIN III, 30-SCC, 30-Adenocarcinoma	Dewaxed formalin-fixed tissue sections	A confocal Raman micro-spectrometer (LabRAM HR Evolution), 532 nm laser source, 25 NM laser power, 50X objective (NA = 0.5)	SVM	Classification of cervicitis, CIN I, CIN II, CIN III, SCC and adenocarcinoma R	[57]

## 4. In Vivo Studies on Cervical Tissues

Table 3 summarises the studies to date on the application of Raman spectroscopy in cervical tissues (in vivo).

In 1998, Mahadevan-Jansen et al. pioneered the use of Raman spectroscopy for the in vivo diagnosis of cervical cancer using a fibre-optic probe [52]. Since then, there have been 11 studies from different parts of the globe where researchers have optimized the acquisition time of the Raman spectrum from 90 s to less than one s. [52,53,54,55,56,57,58,59,60,61,62]. 

In vivo Raman spectroscopy can classify high-grade squamous dysplasia from normal tissue [54] and identify ectocervix, endocervix, and low-grade dysplasia with 95% efficiency [57]. Moreover, the effect of hormonal variation on Raman spectra and its outcome on cervical disease detection was studied [56]. 

The research group in Singapore exhibited that the high wavenumber region of in vivo Raman spectra can detect cervical dysplasia [58]. Another study from the same group illustrates that the concurrent fingerprint and high wavenumber region of Raman spectra has the potential to improve the detection of cervical dysplasia [59], showing that confocal in vivo Raman spectroscopy has the potential to improve early diagnosis of cervical precancer [60]. 

The research group in India evaluated the application of in vivo Raman spectroscopy in the identification of cervical cancers in the Indian population; their group also explored the utility of the vaginal site as an internal control [61]. In addition, the comparative evaluation of diffuse reflectance and Raman spectroscopy was also studied, and their findings suggested that Raman spectroscopy was more efficient than diffuse reflectance spectroscopy in identifying cervical cancer [62].

Although in vivo Raman spectroscopy for cervical cancer is an exciting area, its implementation for in vivo cancer screening has some challenges, such as the logistic requirements for measuring in vivo Raman spectra, such as a dark room, and post-data analysis and patient discomfort. However, it could be a valuable and complementary tool in colposcopy clinics for identifying precancer and cancer patches of tissues during colposcopy surgical procedures. 

**Table 3:** Application of Raman spectroscopy in cervical tissues (in vivo).

Year	Authors	Patient Numbers	Raman Parameter	Data Analysis Methodology	Main Findings	Reference
1998	Mahadevan-Jansen et al. (Richard-Kortum group)	No details given	Laser-789 nm diode laser, laser power 15 mW, spot size 900 µm, an imaging spectrograph and a CCD camera, Fiber-optic probe	Peak ratios	The Raman probe can be used to measure NIR in-vivo Raman Spectra from the cervix, and it suggested that laser power can be up to 80 mW, via Monte Carlo and thermal modelling prediction	[58]
2001	Utzinger et al. (Mahadevan-Jansen group)	13 patients 24 spectra	Laser-789 nm diode laser, laser power 15–16.5 mW, an imaging spectrograph and a CCD camera, Fiber-optic probe-12 mm head, spectral acquisition time-60 to 180 s	Peak ratios	In squamous dysplastic tissue, the ratio of 1454 to 1656 cm^−1^ is higher and 1330 to 1454 cm^−1^ is lower, than in other tissue types	[59]
2007	Robichaux-Viehoever et al. (Mahadevan-Jansen group)	79 patients	Laser-785 nm diode laser, fibre-optic probe (Visionex Inc., Atlanta, GA), an imaging spectrograph, liquid nitrogen cooled charge-coupled device (CCD) camera all controlled with a laptop computer, laser power-80 mW, spectral acquisition time-5 to 15 s	Peak ratios	Raman spectroscopy can distinguish between high-grade dysplasia and benign tissue with 89% sensitivity and 81% specificity	[60]
2009	Kanter et al. (Mahadevan-Jansen group)	43 patients	Laser-785 nm diode laser, fiber optic probe, an imaging spectrograph, thermo-electrically cooled charge-coupled device (CCD) camera, Laser power-80 mW, Penetration depth ~300 um, spectral acquisition time 3 s	MRDF-SMLR	Improvement in the classification efficiency from 88 to 94% by incorporating the hormonal status in the menstrual cycle into the classification algorithm	[61]
2009	Kanter et al. (Mahadevan-Jansen group)	122 patients	Laser-785 nm diode laser, fiber optic probe, imaging spectrograph, thermo-electrically cooled charge coupled device (CCD) camera, Laser power-80 mW, spectral acquisition time 3 s	MRDF-SMLR algorithm	Stratifying the menopause data can improve the classification efficiency of LGSIL to 97% (as compared to previous reports- 74%)	[62]
2009	Kanter et al. (Mahadevan-Jansen group)	66 patients	Laser-785 nm diode laser, fiber optic probe, an imaging spectrograph, thermo-electrically cooled charge-coupled device (CCD) camera, Laser power-80 mW, spectral acquisition time 5 s	LDA, MRDF-SMLR	High-grade spectra classify with 95%, and low-grade spectra classified with 74% classification efficiency, improving classification sensitivity to 98% and specificity to 96%	[63]
2009	Mo et al. (Zhiwei Huang group)	46 patients	Laser-785 nm, a high-throughput spectrometer, equipped with an NIR-enhanced charge-coupled device (CCD) detector, and fiber-optic Raman probe, 100 mW laser power with 1 s acquisition time	PCA-LDA	High wavenumber in vivo Raman Spectroscopy yielded a diagnostic 93.5 % sensitivity and 97.8% specificity.	[64]
2012	Duraipandian et al. (Zhiwei Huang group)	44 patients	Laser-785 nm, a high-throughput spectrometer, equipped with a NIR-enhanced charge-coupled device (CCD) detector, and a custom-made fiber-optic Raman probe with NIR-coated Sapphire ball lens, 100 mW laser power with 1 s acquisition time	PLS-DA	Simultaneous fingerprint and high wavenumber in vivo Raman Spectroscopy can classify dysplasia and normal cervix tissue with 82% classification accuracy.	[65]
2013	Duraipandian et al. (Zhiwei Huang group)	84 patients	Diode laser-785 nm, a high-throughput spectrometer, equipped with a NIR-enhanced charge-coupled device (CCD) detector, and a custom-made fibre-optic Raman probe, 100 mW laser power with 1 s acquisition time	PCA-LDA	84.1% classification accuracy (81% sensitivity and 87% specificity) Best classification achieved using confocal Raman spectroscopy compared to composite NIR AF/Raman spectroscopy or NIR AF spectroscopy alone.	[66]
2014	Rubina et al. (Krishna group)	93 patients	HE-785 commercial Raman spectrometer, diode laser 785, charge-coupled device (CCD) detector, Custom-made Raman Probe, laser power 80 mW and 5-s acquisition time	PCA-LDA	97% classification efficiency for normal and tumour tissue Utility of vaginal tissue as a control	[67]
2016	Rubina et al. (Krishna group)	46 patients	Raman System: HE-785 commercial Raman spectrometer, diode laser 785, charge-coupled device (CCD) detector, Custom-made Raman Probe, laser power 80 mW and 5-s acquisition time Diffuse-reflectance System: Tungsten Halogen lamp, fibre-optic coupled spectrometer, control via laptop, and bifurcated probe (ZR 400-5 VIS/NIR, ocean optics)	PCA-LDA	Classification efficiency of Raman spectroscopy (sensitivity 91%, and specificity 96%) and diffuse reflectance spectroscopy (sensitivity 85%, and specificity 95%).	[68]

## 5. Studies on Biofluids

Table 4 summarises the studies to date on the application of Raman spectroscopy using biofluids for cervical cancer and precancer.

Raman spectroscopy has shown good potential for cervical cancer detection using blood serum or plasma. Feng et al. [69] showed that surface-enhanced Raman spectroscopy (SERS) together with PCA-LDA achieved better sensitivity and specificity than empirical analysis (integration of 1310–1430 and 1560–1700 cm^−1^ spectral bands) in discriminating between plasma from cervical cancer patients and healthy subjects.

Gonzalez-Solis et al. [70] used Raman spectroscopy to discriminate between serum samples from healthy donors and from cervical cancer patients and showed that glutathione, tryptophan, β carotene, and amide III were the main molecular differences. 

Similarly, Shrivastava et al. [71] showed increased levels of glycogen, phenylalanine, tryptophan, amide III, and nucleic acids and decreased levels of glutathione and β carotene in serum from cervical cancer patients compared to healthy donors. During chemoradiotherapy, increases in phospholipids, amide III, and glutathione as well as decreases in proteins and nucleic acids, were observed.

Spectral differences between plasma samples from healthy donors and cervical cancer patients were investigated by Raja et al. [72]. Nucleic acids, protein, tyrosine, and tryptophan were shown to be increased in cervical cancer patients compared to healthy donors, while lipids and β-carotene were higher in healthy donors compared to cervical cancer patients.

Recently, surface-enhanced Raman spectroscopy (SERS) has also been applied to the detection of cervical cancer biomarkers in serum, such as squamous cell carcinoma antigen (SCCA), osteopontin (OPN), cancer antigen 125 (CA125), and surviving [73,74,75] and B7 homolog 6 (B7-H6) protein [76]. SERS-based immunoassays have been developed for simultaneous detection of SCCA and OPN with detection limits of 8.628 pg/mL for SCCA and 4.388 pg/mL for OPN in human serum [73]; for simultaneous detection of SCCA and CA125 with detection limits of 8.093 pg/mL for SCCA and 7.370 pg/mL for CA125 in human serum [74]; and for simultaneous detection of SCCA and surviving with detection limits of 6 pg/mL for SCCA and 5 pg/mL for surviving [75]. In addition, the assays showed good agreement with commercial ELISA kits following the testing of serum samples from healthy donors and patients with cervical cancer and precancer [73,74,75]. 

Furthermore, a SERS-based immunosensor showed detection of the cancer biomarker B7-H6 in cervical cancer patient serum with an improved (100-fold) limit of detection of 10.8 fg/mL compared to a commercial ELISA kit [76].

**Table 4:** Application of Raman spectroscopy in biofluids for cervical cancer.

Year	Authors (research Group)	Sample/Patient Numbers	Sample Type/Sample Prep/Substrate	Raman Parameters	Data Analysis Methodology	Main Findings	Reference
2013	Feng et al. (Zeng group)	110 samples––60 cervical cancer patients, 50 healthy volunteers	After collection and centrifugation, plasma was mixed 1:1 with silver colloidal nanoparticles and incubated for 1.5 h at 4 °C. A drop of plasma on the aluminium plate	Renishaw Raman spectrometer, 785 nm laser 20X objective lens Spectral resolution 2 cm^−1^ 10 s acquisition	Principal Component Analysis Linear Discriminant Analysis (PCA-LDA)	Discrimination of cancer and healthy control serum samples Spectral differences: amino acids, saccharides and esters Sensitivity: 96.7%, Specificity: 92%	[69]
2014	Gonzalez-Solıs et al. (Palomares-Anda group)	42 samples—3 precancer and 19 cervical cancer patients, 20 healthy volunteers	The serum was frozen in liquid nitrogen after collection and centrifugation A drop of serum on aluminium substrate	Jobin-Yvon LabRAM HR800 Raman spectrometer, 830 nm laser, ∼17 mW at the sample 50X objective lens Spectral resolution ∼0.6 cm^−1^ 20 to 40 s acquisition	Principal Component Analysis (PCA)	Discrimination of precancer, cancer, and healthy control serum samples Spectral differences: glutathione, tryptophan, β carotene, and amide III	[70]
2019	Raja et al. (Ganesan group)	48 samples—18 cervical cancer patients and 30 healthy volunteers	Plasma spectra measured in quartz cuvettes immediately after collection and centrifugation	Jobin-Yvon LabRAM HR800 Raman spectrometer, 785 nm laser, ∼12 mW at the sample 60 s acquisition, 2 accumulations	PCA-LDA	Discrimination of cancer and healthy control plasma samples Spectral differences: nucleic acids, protein, tyrosine, tryptophan, lipids, and β-carotene Sensitivity: 94.4%, Specificity: 96.7%	[72]
2020	Lu et al. (Cao group)	150 patient samples—30 healthy subjects, 30 CINI, 30 CINII, 30 CINIII, and 30 cervical cancer patients	Serum frozen at −80 °C after collection and centrifugation SERS tag—Au-Ag nanoshuttles (Au-AgNS) and Capture substrate—hydrophobic filter paper-based Au nanoflowers (AuNF)	No detail given	n/a	SERS-based simultaneous detection of squamous cell carcinoma antigen (SCCA) and osteopontin (OPN) in serum Detection limit: 8.628 pg/mL for SCCA and 4.388 pg/mL for OPN SERS intensities at 1593 cm^−1^ (SCCA) and 1334 cm^−1^ (OPN) increased in serum from cervical cancer and precancer patients compared to healthy subjects Good agreement between SCCA and OPN levels measured by SERS and ELISA in serum samples	[73]
2021	Shrivastava et al. (Singh group)	93 samples—63 cervical cancer patients and 30 healthy volunteers	Serum frozen at −20 °C after collection and centrifugation	WITec Raman alpha 300R Raman microscope, 532 nm laser, ∼28 mW at the sample 50X/0.8 NA objective lens 5 s acquisition, 5 accumulations	PCA-LDA	Discrimination of cancer and healthy control serum samples and of patients at different stages of chemoradiotherapy (before, during, after treatment) Spectral differences: glycogen, phenylalanine, tryptophan, amide III, nucleic acids, glutathione and β carotene (control vs. cancer); phospholipids, glutathione, proteins, and nucleic acids (during chemoradiotherapy) Sensitivity: 50–92.5%, Specificity: 25–85.7%	[71]
2021	Xia et al. (Cao group)	150 patient samples—30 healthy subjects, 30 CINI, 30 CINII, 30 CINIII, and 30 cervical cancer patients	Serum frozen at −80 °C after collection and centrifugation SERS immunoprobes based on monoclonal antibody-coupled and Raman reporter-labeled nano-Ag polydopamine nanospheres (PDA@Ag-NPs)	Renishaw inVia Raman microscope, 785 nm laser 5 mW power 50X objective lens 1 s acquisition	n/a	SERS-based lateral flow assay for simultaneous detection of SCCA and cancer antigen 125 (CA125) in serum Detection limit: 8.093 pg/mL for SCCA and 7.370 pg/mL for CA125 SERS intensities at 1083 cm^−1^ (SCCA) and 1330 cm^−1^ (CA125) increased in serum from cervical cancer and precancer patients compared to healthy subjects Good agreement between SCCA and CA125 levels measured by SERS and ELISA in serum samples	[74]
2021	Liu et al. (Cao/Lu group)	160 patient samples—40 chronic cervicitis, 40 LSIL, 40 HSIL and 40 cervical cancer patients	Serum frozen at −80 °C after collection and centrifugation SERS tag—Au-Ag nanoshells (Au–AgNS) Capture substrate—Au–Ag nanobox (Au–AgNB) array	Raman spectrometer, 785 nm laser 5 mW power 50X objective lens 10 s acquisition	n/a	SERS-based simultaneous detection of SCCA and survivin in serum Detection limit: 6 pg/mL for SCCA and 5 pg/mL for survivin SERS intensities at 1081 cm^−1^ (SCCA) and 1327 cm^−1^ (survivin) increased in serum from cervical cancer and precancer patients compared to patients with cervicitis Good agreement between SCCA and survivin levels measured by SERS and ELISA in serum samples	[75]
2021	Panikar et al. (Del Toro-Arreola/De La Rosa group)	10 patient samples— 9 cervical cancer 1 healthy subject	Serum frozen at −80 °C after collection and centrifugation SERS nanoprobe—anti-B7-H6@ATP@AuNPs Capture substrate -zwitterionic L-cysteine substrate	Renishaw inVia Raman Microscope, 785 nm laser, power 2.4 mW 50X objective lens 20 s acquisition, 10 accumulations	n/a	SERS-based detection of B7-H6 in serum Detection limit: 10.8 fg/mL for B7-H6 SERS intensities at 731 cm^−1^ increased in serum from cervical cancer patients compared to healthy subject Good agreement between B7-H6 levels measured by SERS and ELISA in serum samples and 100-fold increase in SERS detection compared to ELISA for 10^−14^ M B7-H6	[76]

n/a: not applicable; no multivariate analysis carried out in the study.

## 6. Summary and Future Directions

Overall management of cervical cancer has been a great challenge for decades, and it has been managed differently in different countries. Additionally, disease management strategies have changed significantly in the last five years and will undergo further changes in the post-vaccination era. 

As HPV vaccination does not protect against all high-risk HPV types [77,78], more work on multivariant HPV vaccines could help in this area. However, excellent quality screening programmes are still crucial in preventing cervical cancer in this new HPV vaccination era [79,80,81,82]. 

For Raman spectroscopic application on cervical cells in the pre-vaccination era, the majority of studies focused on identifying cancer and precancer cells in the Pap smear, either on cell pellets or single exfoliated cells. In recent years, this work may move more towards identifying HPV-transient or HPV-transforming infection because of the change to HPV primary screening. New methods to help with the triage of HPV-positive women are needed to avoid over-referral to colposcopy clinics. Additionally, surface-enhanced Raman spectroscopy (SERS) based biosensing of the early biomarkers using minimally invasive samples such as biofluids (blood, urine) or exfoliated cells on larger patient cohorts is required, as SERS offers the potential possibility of a point-of-care testing.

In the domain of diagnosis and prevention, in vivo based Raman spectroscopically guided colposcopy would be an exciting area to further explore in larger cohorts to improve the accuracy of the current colposcopy-guided primary interventions. Additionally, response to radiation and/or chemotherapy could be interesting areas to explore via minimally invasive approaches e.g., exfoliated cells or biofluids. 

## Data Availability

Not applicable.
